# Stamping Nanoparticles onto the Electrode for Rapid Electrochemical Analysis in Microfluidics

**DOI:** 10.3390/mi12010060

**Published:** 2021-01-06

**Authors:** Jiyoung Son, Edgar C. Buck, Shawn L. Riechers, Xiao-Ying Yu

**Affiliations:** Energy and Environment Directorate, Pacific Northwest National Laboratory, Richland, WA 99354, USA; jiyoung.son@pnnl.gov (J.S.); edgar.buck@pnnl.gov (E.C.B.); shawn.riechers@pnnl.gov (S.L.R.)

**Keywords:** nanoparticle, working electrode, microfluidic electrochemical cell, epoxy stamping, conductive epoxy, CeO_2_, graphite

## Abstract

Electrochemical analysis is an efficient way to study various materials. However, nanoparticles are challenging due to the difficulty in fabricating a uniform electrode containing nanoparticles. We developed novel approaches to incorporate nanoparticles as a working electrode (WE) in a three-electrode microfluidic electrochemical cell. Specifically, conductive epoxy was used as a medium for direct application of nanoparticles onto the electrode surface. Three approaches in this work were illustrated, including sequence stamping, mix stamping, and droplet stamping. Shadow masking was used to form the conductive structure in the WE surface on a thin silicon nitride (SiN) membrane. Two types of nanomaterials, namely cerium oxide (CeO_2_) and graphite, were chosen as representative nanoparticles. The as-fabricated electrodes with attached particles were characterized using atomic force microscopy (AFM) and time-of-flight secondary ion mass spectrometry (ToF-SIMS). Electrochemical analysis was performed to verify the feasibility of these nanoparticles as electrodes. Nanomaterials can be quickly assessed for their electrochemical properties using these new electrode fabrication methods in a microfluidic cell, offering a passport for rapid nanomaterial electrochemical analysis in the future.

## 1. Introduction

Electrochemical analysis of inorganic, metal, or metal alloy in the nanoparticle size range is a valuable tool to evaluate, identify, or determine unique properties of novel materials in diverse applications [1,2,3,4,5]. Cyclic voltammetry (CV) is an electrochemical method that measures the current in an electrochemical cell while cycling the potential of the working electrode (WE) [6]. Microfluidic electrochemical cells enable similar CV studies of micro- and nanomaterials [7,8,9].

A number of electrochemical analysis methods have been used to study powdered materials that have been deposited directly on an electrode surface consisting of polymer composites, graphite, and silver [5,10,11,12]. These applied materials were covered with either a thick-film deposit or clay pastes. Earlier studies were limited due to fragility and poor reproducibility of the electrode as a result of the low conductivity of polymer composites, zeolites, and clays. Additionally, high current and resistance of the thick-film deposits can be compounded by high current signals coming from reactions of the materials. Controlling the amount of the material dispensed onto a surface has been difficult in the process of direct deposition on an electrode (DDE) [1,10].

Our group at the Pacific Northeast National Laboratory (PNNL) recently developed a microfluidic device for multimodal spectroscopy and microscopy of liquids [13,14]. This vacuum compatible device is named System for Analysis at the Liquid-Vacuum Interface (SALVI). The electrochemical SALVI, or SALVI E-cell, contains three electrodes and allows simultaneous electrochemical analysis coupled with in operando spectroscopy and microscopy of chemical imaging tools such as time-of-flight secondary ion mass spectrometry (ToF-SIMS) and scanning electron microscopy (SEM) [15,16]. This technology provides the potential to investigate the electrochemical behavior of various materials [15,17,18]. The basic configuration of SALVI E-cell was used to test and verify the novel nanoparticle stamping methods to fabricate working electrodes (WEs) in this study.

**Figure 1:**
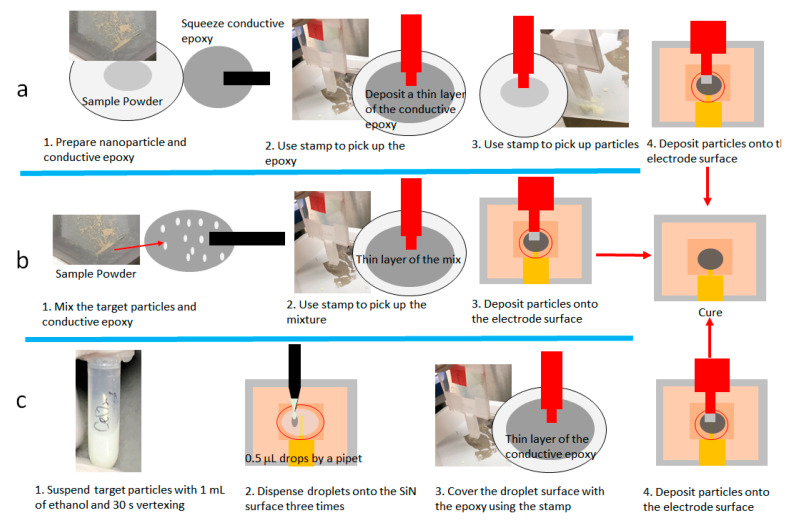
Overview of three different silver conductive epoxy stamping methods for depositing nanoparticles onto the electrode: (**a**) sequence stamping; (**b**) mix stamping; and (**c**) droplet stamping.

We developed a new approach to incorporate nanoparticles onto the WE of the SALVI E-cell to solve the known issues of DDE. The technique utilizes the silver conductive epoxy stamping method on the conductive layer to form an effective WE that contains nanoparticles. Epoxy stamping has been previously used in electronic component packaging [19]; however, to the best of our knowledge, stamping has not been used for making electrodes. Three different methods to prepare electrodes containing nanoparticles were compared in this work, including sequence stamping, mix stamping, and droplet stamping as shown schematically in Figure 1. All three methods used a stamped epoxy layer to protect the nanoparticles from being washed off by the electrolyte solution. The particle inclusion steps vary, and their effectiveness was compared in this investigation. 

Two representative types of nanoparticles, namely, cerium oxide (CeO_2_) and graphite, were selected to show the potential range of the new approach and demonstrate feasibility. Both have been widely used nanomaterials for energy storage systems and sensor materials [20,21,22,23,24,25,26,27]. Six types of SALVI E-cells were fabricated for CV analysis and device performance comparison. CV plots provide unique electrochemical current reaction profiles of each device fabricated, using different nanoparticle stamping methods for CeO_2_ and graphite, respectively. Additionally, SALVI E-cells without any nanoparticles in the WE were used as a control. Atomic force microscopy (AFM) was used to characterize nanoparticle morphology on the WE surface. ToF-SIMS, a powerful imaging mass spectrometry for surface analysis [28], was used to acquire chemical maps of CeO_2_ and graphite on the stamped WE surface to evaluate electrode fabrication effectiveness.

## 2. Materials and Methods

Two nanoparticles, cerium oxide (CeO_2_, US research materials Inc., Houston, TX, USA, 10 nm mean diameter) and graphite (Graphite NanoPlatelets, Lansing, MI, USA, xGnP, 10–15 nm mean diameter) were used. The existing SALVI E-cell has a gold thin film WE sputter coated on the back side of the silicon nitride (SiN) window (Norcada, 0.5 × 0.5 mm on a 200 µm Si frame). Electrode deposition was done by the following steps: sputtering a layer of titanium of 10 nm as the adhesion layer, followed with a 40 nm gold conductive layer on the SiN substrate. Conductive epoxy (Silver conductive epoxy, Chemtronics, Kennesaw, GA, USA) was used for stamping and applying nanoparticles onto the electrode surface. Stamps were fabricated from 1.5 mm thick polymethyl methacrylate (PMMA) sheets and a CO_2_ laser cutter (H-series, Full Spectrum Laser, Las Vegas, NV, USA). Ethanol was used as a carrier liquid for droplet spraying application. Nanoparticle handling was performed under a ventilated nano enclosure (XPert Nano Enclosure, LABCONCO, Kansas City, MO, USA). 

### 2.1. Nanoparticle Electrode Fabrication and SALVI E-Cell Fabrication

Figure 1 depicts the schematic of nanoparticle stamping. Handling of CeO_2_ and graphite nanoparticles followed the same workflow. Specifically, nanoparticles were applied directly on the boundary of the gold coated SiN surface using stamping techniques. Three stamping techniques: (1) sequence stamping, (2) mixing and stamping, and (3) droplet spraying and stamping were developed and compared. The SALVI device fabrication was conducted in a clean room to prevent particle contamination from the electrode surface after making WE electrodes using stamping in a nanoenclosure. Further details of the E-cell fabrication were reported elsewhere [15,17]. The use of SiN was not imperative in the electrochemical cell fabrication. The main reason to use SiN is to make it compatible with other in situ spectroscopy, optical microscopy, and electron microscopy tools. Thus, SiN was not a prerequisite substrate in stamping.

#### 2.1.1. Sequence Stamping (Sequence Stamp) 

A thin layer of silver conductive epoxy was squeezed in a plate in sequence stamping as the first method. A custom-made stamping tool with a stamping surface of 1.5 mm × 1.5 mm was used to pick up a thin layer of ~200 µm of the silver epoxy onto the stamping surface. Next, ~0.3 mg nanoparticles were picked up by the uncured epoxy on the tip of the stamping surface. Lastly, the epoxy layer with particles gently touched the gold conductive layer to leave the nanoparticles on the gold coated surface as WE. Stamped SiN windows were cured for 1 h in the nano enclosure.

#### 2.1.2. Mixing and Stamping (Mix Stamp) 

The second method used conductive epoxy to mix and incorporate nanoparticles and form a nanoparticle mix before stamping (Figure 1b). First, ~3 mg nanoparticles of graphite and 30 mg CeO_2_ were spread gently on top of a thin layer of ~100 mg conductive epoxy, respectively, in a plastic plate. Additional conductive epoxy (~50 mg) was added to make a consistent mixture. The mixture was smeared into a thin layer on a clean surface. Next, the nanoparticle-containing epoxy layer was picked up by a custom-made PMMA stamp. The final step included gently smearing the stamp surface onto the gold conductive layer as in sequence stamping. Stamped SiN windows were set under the nano enclosure for 1 h curing. 

#### 2.1.3. Droplet Spraying and Stamping (Droplet Stamp) 

The droplet spraying and stamping method used ethanol droplets as a nanoparticle carrier to deposit onto the conductive gold layer on the substrate (Figure 1c). The spraying liquids were prepared with 3 mg of graphite or 30 mg CeO_2_ in 1 mL of ethanol in plastic tubes. Particles were well suspended by 1 min vertexing and 15 min sonication. Then 3 µL of the liquid mixture was dropped on the substrate using a pipette. Particles were distributed on the substrate upon ethanol evaporation. The final step of epoxy stamping was to cover the electrode with a conductive epoxy glue layer, same as the other two methods. 

### 2.2. Electrochemical Analysis

**Figure 2:**
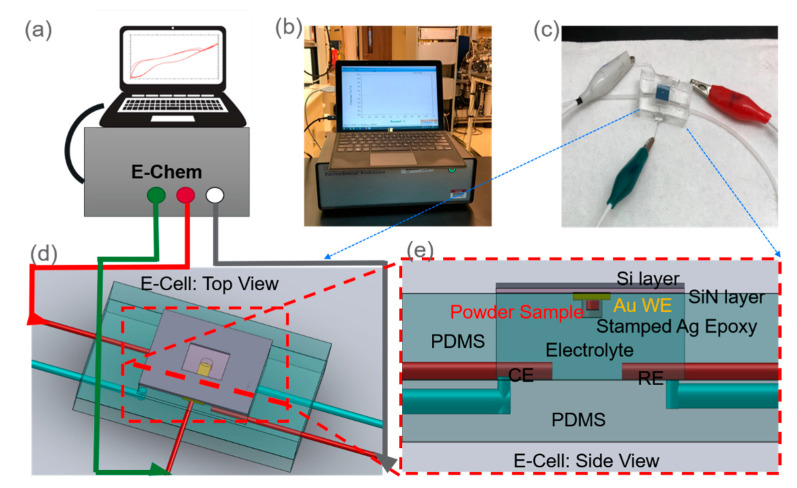
The overview of the electrochemical analysis setup: (**a**) the electrochemical station with the control computer green port (WE), red port (CE), and white port (RE); (**b**) a picture of the electrochemical station; (**c**) a close-up picture of the SALVI E-cell connected with alligator clips to the electrochemical station; (**d**) a 3D rendering image of the top view of the SALVI E-cell; and (**e**) a cross-section view of the microfluidic cell.

Three types of E-cell devices were tested for cerium oxide and graphite particles, respectively, each representing different nanoparticle inclusion methods. Two control devices were prepared for comparison as well. One had only a silver epoxy stamped layer as the WE and the other had no epoxy stamping at all. In total, eight devices were tested. An electrochemical station (CH Instruments, Austin, TX, USA, 660 h) was used (Figure 2b). The following scan rates were used 10, 20, 40, 60, 80, and 100 mV/s, respectively, for each device. The sensitivity range was set at 1 × 10^−4^–10 × 10^−5^ A/V. The voltages of sweeping polarity direction ranged from 1 V to −1 V then reverse back to 1 V. There were at least 20 sweeps at each scanning rate. The 2 mM potassium thiocyanate (KSCN) solution was used as the electrolyte [29]. The 0.1 mL of KSCN solution was injected at the rate of 100 µL/min into the microfluidic device using a syringe pump (Cole-Parmer, Vernon-Hills, IL, USA, model 270) prior to analysis. Known CV procedures were followed [6]. Collected CV data were exported to Origin Pro (2017) for plotting.

### 2.3. Optical Microscopy of WE and AFM Characterization

An optical microscope (VHX500, Keyence, Osaka, Japan) was used to image the surface of CeO_2_ and graphite nanoparticles after they were applied onto the substrate. Optical images were recorded with the built-in camera with at a magnification of 50. Topographical analysis also was performed using an MFP-3D Infinity AFM (Asylum, Oxford, UK). Tapping mode measurements were performed using an etched silicon probe (Bruker, Billerica, MA, USA, RTESPA-300, 8 nm nominal tip radius, 40 N/m spring constant) with a set point of 0.8 V and a scan speed of 1 Hz. AFM images were leveled using the flat portion of the substrate, which was used as a baseline to determine the height of individual particles. Samples for AFM imaging were prepared by suspending nanoparticles using 1.5 mL ethanol with a ratio of nanoparticles to ethanol of 1 to 5. Samples were sonicated for 3 h. Five µL of the suspended nanoparticle sample was deposited on a freshly cleaved mica surface (1.5 cm × 1.5 cm) and let dry for 20 min. The dried mica surface was blasted twice with pure nitrogen before analysis.

#### ToF-SIMS Characterization of WE

Chemical maps of the as-fabricated WE surfaces were collected using a ToF-SIMS instrument (IONTOF GmbH, ToF-SIMS V, Münster, Germany). The pressure of the main chamber was maintained at 1 × 10^−8^ mbar during analysis. The primary ion beam was a 25 keV Bi_3_^+^ with 10 kHz pulse energy. The pulse width was 0.8 ns and the current was ~0.6 pA. ToF-SIMS 2D images were acquired by rastering over an area of 500 × 500 μm^2^ for 100 scans. ToF-SIMS high resolution spectral data were acquired by rastering over an area of 500 × 500 μm^2^ for 60 scans. The ToF-SIMS data was processed using IONTOF Surface Lab 7.0 software. The mass spectra were calibrated by using peaks, including C (m/z^+^ 12.0), CH_3_ (m/z^+^ 15.02), Na (m/z^+^ 22.98), C_4_H_9_ (m/z^+^ 57.07), Ag (m/z^+^ 106.90), and CeO (m/z^+^ 155.90).

## 3. Results

**Figure 3:**
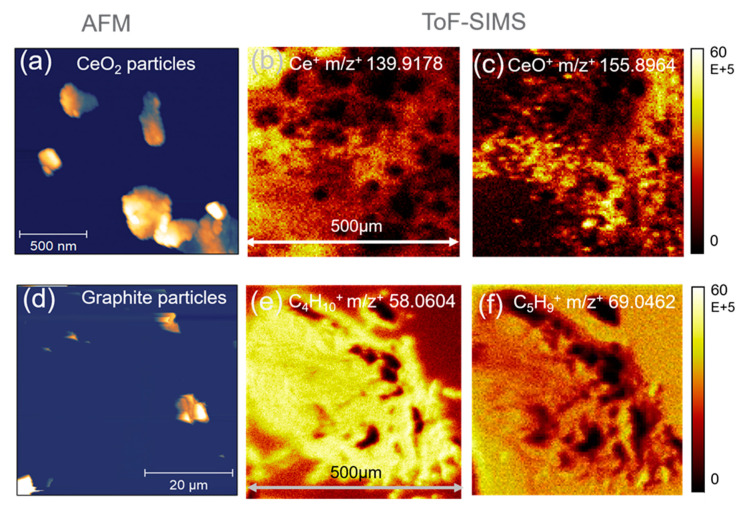
AFM and ToF-SIMS 2D mapping of the working electrodes containing nanoparticles: (**a**) AFM image of CeO_2_; ToF-SIMS 2D mapping of Ce^+^ (**b**) and CeO^+^ (**c**) on the as-made working electrode (WE) using CeO_2_ sequence stamping; similarly (**d**) AFM image of graphite; ToF-SIMS 2D mapping of C_4_H_10_^+^ (**e**) and C_5_H_9_^+^ (**f**) on the as-made WE using graphite mixing and stamping.

A series of experiments were conducted to verify the effectiveness of nanoparticle DDE in WE fabrication. As shown in Figure 3, optical and AFM images show nanoparticle distribution and as-deposited conditions on the substrate. The ToF-SIMS 2D mapping and spectral data give evidence that nanoparticles are present on the substrate surface. The CV scans contain unique signatures of the redox peaks showing the validity of WE performance. Single device electrochemical reproducibility and device-to-device reproducibility data are presented in Figures S8–S13. Our results show consistent performance of the three methods, respectively, concerning electrochemical performance at different scanning rates. The device-to-device reproducibility can be improved by depositing the same amount of nanoparticles onto the surface.

### 3.1. Optical Microscopy and AFM Characterization

**Figure 4:**
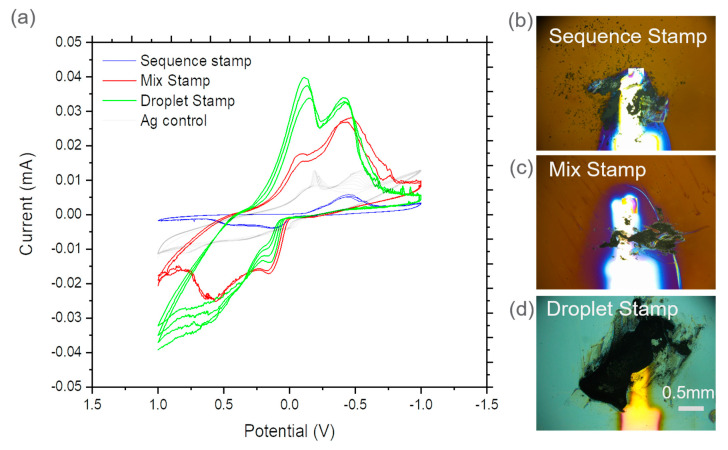
(**a**) CV comparisons of WE surfaces containing CeO_2_ nanoparticles using three stamping methods and the control device and optical microscope image of (**b**) sequence stamping, (**c**) mixing and stamping; and (**d**) droplet spraying and stamping. The shining finger-like feature in (**b**–**d**) is the Au conductive layer.

**Figure 5:**
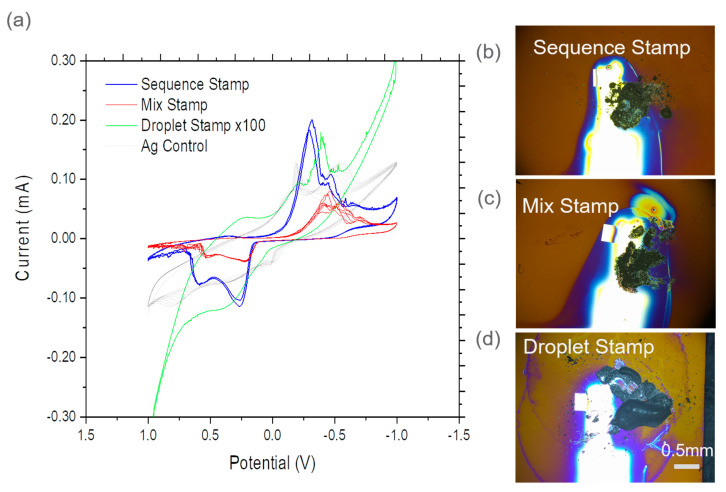
(**a**) CV comparisons of WE surfaces containing graphite nanoparticles using three stamping methods and the control device and optical microscope image of (**b**) sequence stamping, (**c**) mixing and stamping; and (**d**) droplet spraying and stamping. The shining finger-like feature in (**b**–**d**) is the Au conductive layer.

Figure 4b–d and Figure 5b–d show optical microscopy images of as-made WE surfaces using three methods and two types of nanoparticles. The size of stamped electrode surface morphology is similar to that of the PMMA stamping tool. However, images had some irregularity in the morphological profile. Figure 3a,d depict nanoparticles deposited on substrates using AFM imaging. Particles appeared to coagulate, for instance, in the spraying and stamping approach. Figure 5a shows CeO_2_ clusters of approximately 500 nm in size. This is significantly bigger than the known 10 nm average particle size. Figure 5d also shows graphite clusters of ~10 µm, which is significantly bigger than the original 10–15 nm particle size prior to fabrication.

### 3.2. ToF-SIMS 2D Mapping and Spectral Analysis

Figure 3 shows ToF-SIMS chemical 2D mapping of stamped electrode surfaces. Figure 3b,c show 2D maps of the CeO_2_ sequence stamped surface. Strong Ce ^+^ m/z^+^ 139.9178 ion (Figure 3b) and CeO^+^ m/z^+^ 155.8964 ion mapping (Figure 3c) were observed. Additionally, these two peaks (Ce^+^ and CeO^+^) had a high abundance in the ToF-SIMS spectra (Figure S1a). Both results support that CeO_2_ particles were successfully incorporated in the electrode surface.

Similarly, Figure 3e,f are ToF-SIMS 2D maps of hydrocarbon fragment peaks C_4_H_10_^+^ m/z^+^ 58.0604 and C_5_H_9_^+^ m/z^+^ 69.0462 from the graphite mixing and stamping WE surface. Identification of these fragments as graphite fragments has been reported previously [30,31]. Additional SIMS spectral plots show dominant counts of these two peaks in supporting information (SI) Figure S1b. Additionally, SIMS 2D mapping and spectral results of the other four types of as-fabricated WE surfaces were presented in the Supplementary Materials in Figures S2–S5.

### 3.3. CV of Nanoparticle Stamped WE

Figure 4 and Figure 5 show CV comparisons among three different stamping methods (e.g., sequence stamping, mix stamping, droplet stamping) for CeO_2_ and graphite nanoparticles as WEs, respectively. The CV results from the epoxy control device as a reference point was also included. For example, the blue cyclic voltammagrams represent the sequence stamping results, red mix stamping, green droplet stamping, and gray results from the silver epoxy control device. The scan rate was 100 mV/s. Additional CV results of control devices can be found in the Supplementary Materials (Figures S6 and S7).

Overlapped plots provide ease of comparison to interpret CV results from different CeO_2_ devices. During potential sweeping from 1 V to −1 V, two peaks were observed in the mix stamping and droplet stamping devices, both shifting toward 0 V in Figure 4a. In the mix stamping device, the double peaks appeared at −0.05 V and −0.22 V. In the droplet stamping device, the double peaks appeared at −0.12 and −0.42 V. The sequence stamping device has a peak at −0.4 V during the 1 V to −1 V sweep. During the reverse direction potential sweeping from −1 V to 1 V, peaks from mix stamping and droplet stamping devices appeared at 0.2 V and 0.55 V; while the silver epoxy device had two peaks at 0.03 V and 0.3 V. Peaks from the sequence stamping device were 0.1 V and 0.45 V. The unique peaks observed here are related to CeO_2_ reduction. The peaks at 0.1 V and 0.45 V were reported previously in CeO_2_ electrochemical analysis [32]. This agreement provides verification of the WE performance.

The graphite device comparisons are depicted in Figure 5a. During the potential sweep from 1 V to −1 V, a single major peak appeared between −0.3 V to −0.5 V; while double peaks appeared in the silver epoxy control device. There were a major peak at −0.3 V and two minor peaks at 0.36 V and −0.46 V from the sequence stamping device. In the mix stamping device, two major peaks appeared at −0.42 V and −0.55 V and a minor peak at 0.3 V. In the droplet stamping device, there was a major peak at −0.42 V and a minor peak at 0.22 V. In the reverse direction potential sweep from −1 V to 1 V, two peaks appeared in the sequence stamping and mix stamping devices, while the epoxy control had three minor peaks at −0.47 V, 0.78 V, and 0.017 V. During the reverse potential sweep, sequence stamping showed major peaks at 0.26 V and 0.6 V and a minor peak appeared at −0.5 V. Mix stamping showed a minor peak at −0.54 V and two major peaks at 0.22, and 0.53 V. Droplet stamping had a minor peak at −0.42 V and a major peak at 0.35 V. Overall, characteristic peaks of graphite redox reaction appear in two stamping methods used to prepare WEs. However, these peaks do not appear in the epoxy control device. The observed peaks from the graphite CV sweeps show similarities to a previously reported peak at 0.22 V in graphite electrochemical analysis [25,33].

## 4. Discussion

Vacuum compatibility of the completed devices was not illustrated in this work. Because the SALVI platform was originally developed for multimodal imaging in vacuum surface instrumentations, the next step will be vacuum compatibility testing prior to in operando analysis. The completed devices using the established SALVI procedure should allow SEM and ToF-SIMS analysis. The vacuum compatibility verification will be presented in the future.

ToF-SIMS 2D maps and spectral results verify that CeO_2_ and graphite nanoparticles were deposited onto the substrate surface using three methods involving stamping. The irregularities in the surface morphology of stamped surfaces shown in Figure 4b–d and Figure 5b–d were likely caused by the multiple smears of stamping layers. This process can be improved by using single stroke stamping in the next phase of development. The stamp tip can be fabricated with more flexible material such as polyurethane instead of PMMA. This change can be helpful to make a soft and flexible stamping contact motion, which will lead to smoother stamping surfaces after controllable single strokes. The new tip will be slightly bigger than the footprint of SiN membranes, which could be used to make controlled stamping and deposition onto the SiN window to form WE. Additionally, leaving the particles in the nano enclosure for 20–30 min before handling sample particles could give better stamping results due to reduced electrostatic particle interaction.

The sequence stamping plot shows different profile characteristic from mixing and droplet approaches followed with stamping (e.g., in Figure 4). Droplet spraying and stamping seems to be most effective to introduce CeO_2_ nanoparticles onto the conductive substrate as an electrode compared to the other two means. Figure 5a shows that droplet spraying and stamping have different profiles. This disagreement may be caused by the difficulty in weighing a small number of nanoparticles and applying the silver epoxy onto the electrode without high accuracy. Among the three tested methods, sequence stamping is the most effective technique for capturing fluffy graphite particles and introducing them onto the substrate. A solution to improve device consistency is to control the amount of powder applied to the substrate. The sample contact surfaces of the PMMA stamping tool can be decreased and upgraded with a more flexible material, such as the polyurethane stamping tip, as discussed earlier. The inconsistency issue also can be minimized when this method is used to handle coarser particles instead of nanoparticles, because the latter are light and fluffy with high electrostatics. It is important that the particle size and amount be controlled and deposited in a well-defined area for optimal electrochemical analysis results regardless of particle size.

The illustrated stamping methods provide an alternative solution compared to DDE reported previously [1,10], because these new ones have better control in applying a small number of nanoparticles onto a substrate, that minimized signals from background reactions. The applied amount can be controlled by altering stamping footprint size depending on the materials.

CeO_2_ nanoparticles are being developed to improve cathodes for energy storage systems via microwave and thermal techniques [20,34]. CeO_2_ is also being used as an analogue for studying uranium oxide corrosion [35]. Electrochemical pretreatment with graphite nanoparticles also has been actively studied to develop more efficient sensors and electrodes [25,26]. Therefore, methods introduced in this paper can potentially provide a new passport to analyze nanomaterials. This is especially important and powerful for assessing characteristics and electrochemical performance of newly synthesized materials.

## 5. Conclusions

A new epoxy stamping technique has been developed to enable rapid electrochemical analysis of nanoparticles. Two representative nanoparticles (CeO_2_, graphite) are stamped directly onto the thin film conductive gold conductive layer of an established microfluidic electrochemical cell as the WE in a three-electrode system. Silver conductive epoxy acts as an adhesive agent for nanoparticles in the stamping method. Three different kinds of stamping methods, including sequence, mix, and droplet stamping, were compared for WE performance in the SALVI E-cells. The particle distribution on the WE surface was verified using AFM and the particle chemical composition was characterized using ToF-SIMS to validate particle attachment. Cyclic voltammagrams of devices using the above stamping methods successfully demonstrated the unique electrochemical profiles of the nanoparticles. Since it is difficult to pick up the same amount of particles using stamping techniques, additional modification is needed to improve these approaches. Our results showed that epoxy stamping can be a useful method for rapid analysis of nanoparticles to determine their electrochemical properties using microfluidics.

## Supplementary Materials

The following are available online at https://www.mdpi.com/2072-666X/12/1/60/s1, Figure S1: ToF-SIMS Spectral plots from the stamped SiN window, Figure S2: ToF-SIMS 2D images and a spectrum plot of the CeO2 mix stamped on SiN membrane, Figure S3: ToF-SIMS 2D images and a spectrum plot of the CeO2 droplet stamped on SiN membrane, SIMS 2D image of Ce+, Figure S4: ToF-SIMS 2D images and a spectrum plot of the graphite sequence stamped on SiN membrane, SIMS 2D image of the C4H10+, Figure S5: ToF-SIMS 2D images and a spectrum plot of the graphite droplet stamped on SiN membrane, Figure S6: Optical image of silver epoxy stamped SiN membrane and Cyclic voltammetry scan of the silver epoxy control device, Figure S7: Optical image of blank SiN membrane with Au electrode and CV scan of the blank control device, Figure S8: CV scans of a sequence stamp device with different scan rates, Figure S9: Comparison of CV scan of the two sequence stamp devices using the scan rate of 100 mV/s Figure S10: CV scans of a mix stamp device with different scan rates, Figure S11: Comparison of CV scan of the two mix stamp devices using the scan rate of 100 mV/s, Figure S12: CV scans of a droplet stamp device with different scan rates, Figure S13: Comparison of CV scan of the two droplet stamp devices using the scan rate of 100 mV/s.
