# Supplementary material for: Stamping Nanoparticles onto the Electrode for Rapid Electrochemical Analysis in Microfluidics
Source: Micromachines (Basel). 2021 Jan 6;12(1):60. doi: 10.3390/mi12010060 (PMC7825540; doi:10.3390/mi12010060)
Supplement: Supplementary file 1 [file micromachines-12-00060-s001.pdf]

Stamping nanoparticles onto the electrode for rapid electrochemical analysis in microfluidics

Jiyoung Son[1], Edgar Buck,[2] Shawn Riechers,[2], and Xiao-Ying Yu[1,*]

[1] Energy and Environment Directorate, Pacific Northwest National Laboratory, Richland, WA 99354; jiyoung.son@ pnnl.gov, edgar.buck@pnnl.gov, shawn.riechers@pnnl.gov, xiaoying.yu@pnnl.gov

[2*] Correspondence: xiaoying.yu@ pnnl.gov; Tel.: +01-509-372-4524 (X.-Y. Y.)

Table of Contents

Supplemental Figures S-1

Figure. S1. ToF-SIMS Spectral plots from the stamped SiN window: (a) surface spectrum of the SiN membrane with CeO_2 sequence stamping and (b) surface spectrum of the SiN membrane with graphite applied by mixed stamping. S-1

Figure. S2. ToF-SIMS 2D images and a spectrum plot of the CeO_2 mix stamped on SiN membrane, (**a**) SIMS 2D image of the Ce^+, (**b**) CeO^+, and (**c**) spectral plot showing Ce^+ ion related peaks S-2

Figure S3. ToF-SIMS 2D images and a spectrum plot of the CeO_2 droplet stamped on SiN membrane, SIMS 2D image of Ce^+(**a**) spectral plot showing Ce^+ ion related peaks. S-3

Figure. S4. ToF-SIMS 2D images and a spectrum plot of the graphite sequence stamped on SiN membrane, SIMS 2D image of the $C_4H_{10}^+$ (**a**) and $C_5H_9^+$ (**b**), and (**c**) spectral plot showing graphite fragment related peaks. S-4

Figure. S5. ToF-SIMS 2D images and a spectrum plot of the graphite droplet stamped on SiN membrane, (**a**) SIMS 2D image of the $C_4H_{10}^+$ and (**b**) $C_5H_9^+$, and (**c**) spectral plot showing graphite fragment ion related peaks. S-5

Figure. S6. Optical image of silver epoxy stamped SiN membrane and Cyclic Voltammetry (CV) scan of the silver epoxy control device of 50 x (**a**) and 200 x (**b**) magnification, and (**c**) CV scan plot. S-6

Figure. S7. Optical image of blank SiN membrane with Au electrode and CV scan of the blank control device of 50 x (**a**) and 200 x (**b**) magnification, and (**c**) CV scan plot. S-7

Figure. S8. CV scans of a sequence stamp device with different scan rates, i.e., 40 mV/s, 80 mV/s, and 100 mV/s. S-8

Figure. S9. Comparison of CV scan of the two sequence stamp devices using the scan rate of 100 mV/s: (**a**) the same device as shown in the main test and (**b**) a different device. S-8

Figure. S10. CV scans of a mix stamp device with different scan rates, i.e., 60 mV/s, 80 mV/s, and 100 mV/s. S-9

Figure. S11. Comparison of CV scan of the two mix stamp devices using the scan rate of 100 mV/s: (**a**) the same device as shown in the main test and (**b**) a different device. S-9

Figure. S12. CV scans of a droplet stamp device with different scan rates, i.e., 60 mV/s, 80 mV/s, and 100 mV/s. S-10

Figure. S13. Comparison of CV scan of the two droplet stamp devices using the scan rate of 100 mV/s: (**a**) the same device as shown in the main test and (**b**) a different device. S-10

Additional experimental details, figures, and tables are provided to support the content in the main text.

Supplemental Figures

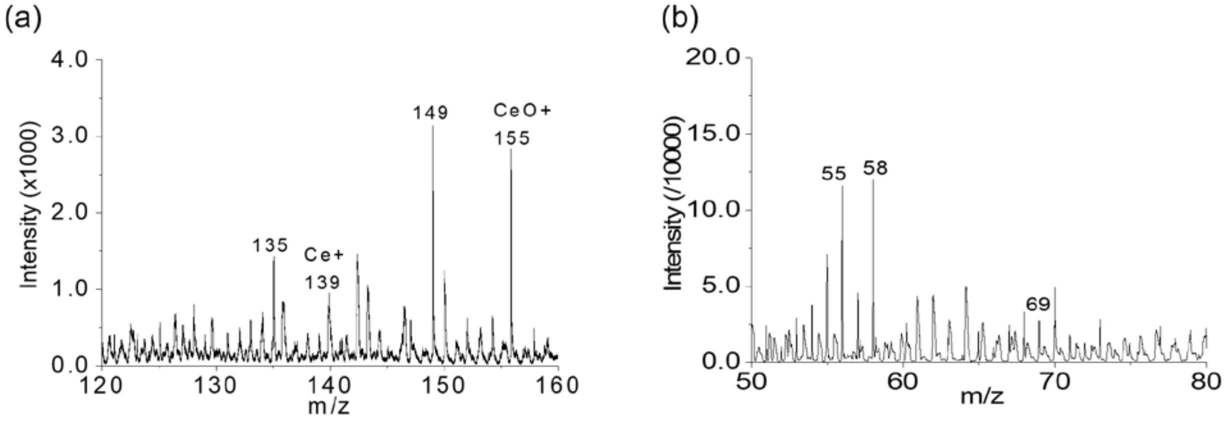

Figure. S1. ToF-SIMS Spectral plots from the stamped SiN window: (a) surface spectrum of the SiN membrane with CeO_2 sequence stamping and (b) surface spectrum of the SiN membrane with graphite applied by mixed stamping.

Figure S1 depicts ToF-SIMS spectral showing mass fragment peaks of targeted nanoparticles, Ce^+ m/z^+ 139.9178 and CeO^+ m/z^+ 155.8964 show cerium fragment peaks from the CeO_2 nanoparticle (Figure S1a). Fewer significant numbers were used in the spectra due to space constraints. Figure S1b shows graphite fragment peaks $C_4H_{10}^+$ m/z^+ 58.0604 and $C_5H_8^+$ m/z^+ 69.0462, and both are previously reported in graphite electrode studies [1, 2].

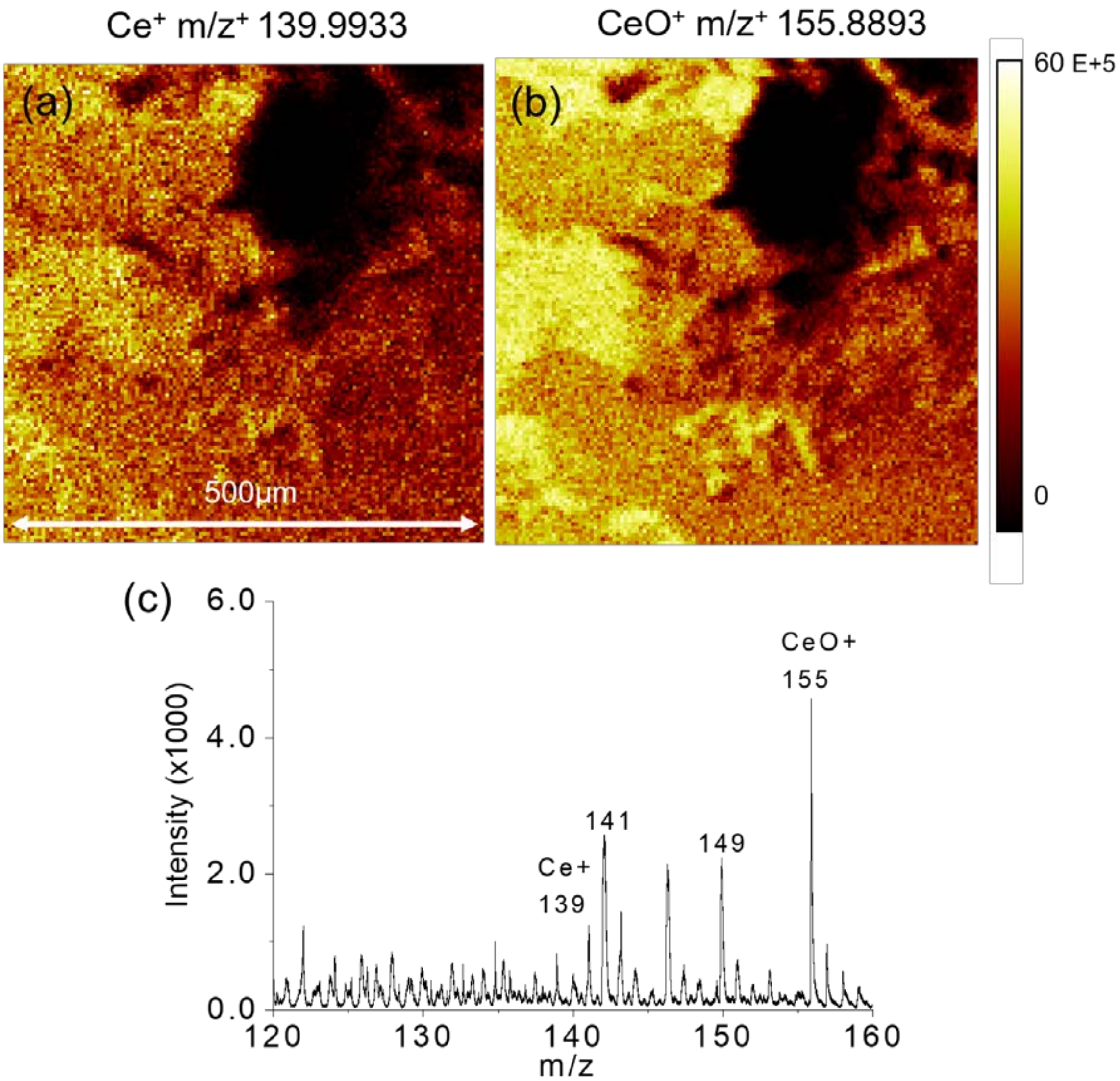

Figure. S2. ToF-SIMS 2D images and a spectrum plot of the CeO_2 mix stamped on SiN membrane, (**a**) SIMS 2D image of the Ce^+, (**b**) CeO^+, and (**c**) spectral plot showing Ce^+ ion related peaks.

Fewer significant numbers were used in the spectra due to space constraints.

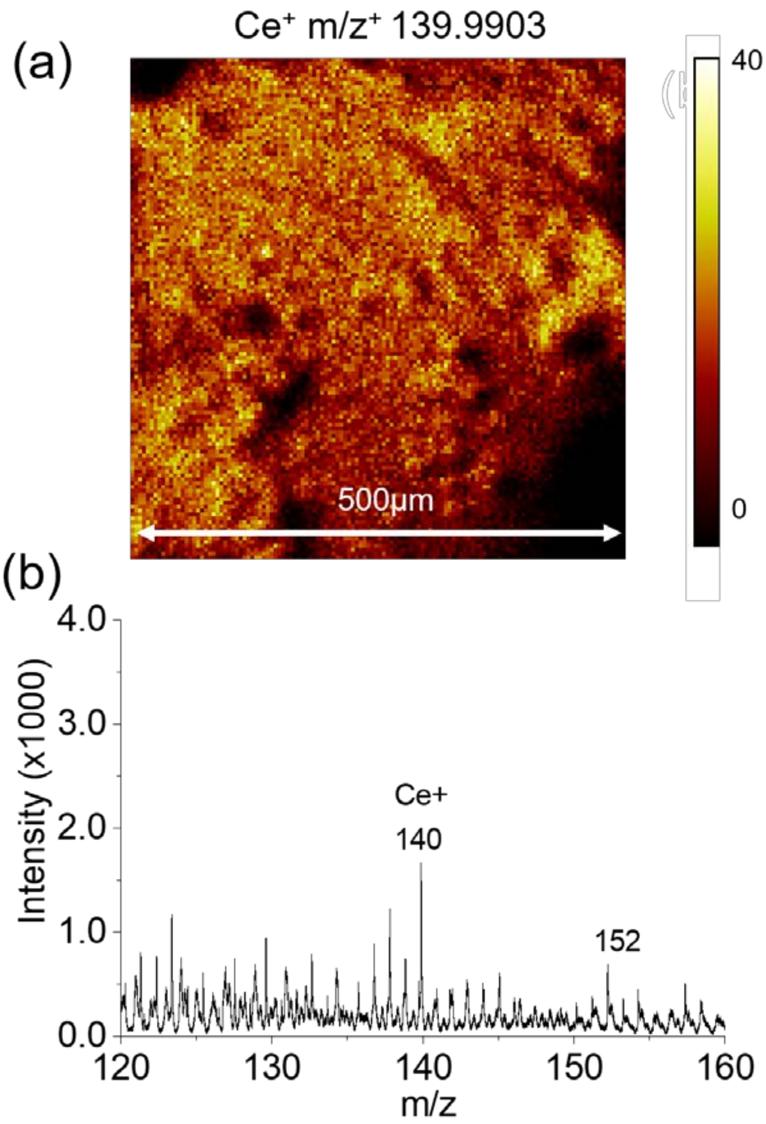

Figure S3. ToF-SIMS 2D images and a spectrum plot of the CeO_2 droplet stamped on SiN membrane, SIMS 2D image of Ce^+(**a**) spectral plot showing Ce^+ ion related peaks.

Note that the Ce^+ m/z^+ 139.9178 peak has different ion counts as Ce^+ in the Figure S3c spectral plot because the sample surface is not as smooth. Fewer significant numbers were used in the spectra due to space constraints.

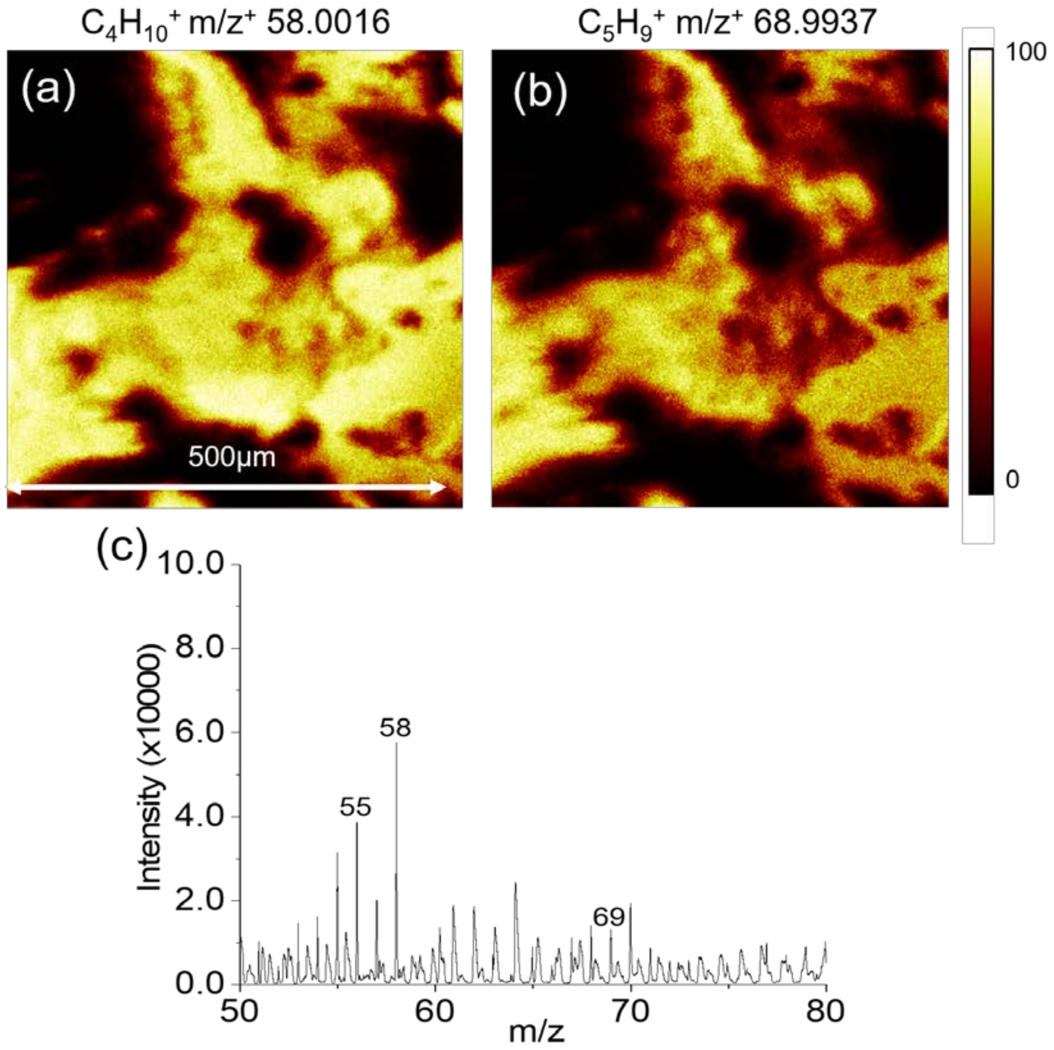

Figure. S4. ToF-SIMS 2D images and a spectrum plot of the graphite sequence stamped on SiN membrane, SIMS 2D image of the $C_4H_{10}^+$ (**a**) and $C_5H_9^+$ (**b**), and (**c**) spectral plot showing graphite fragment related peaks.

Fewer significant numbers were used in the spectra due to space constraints.

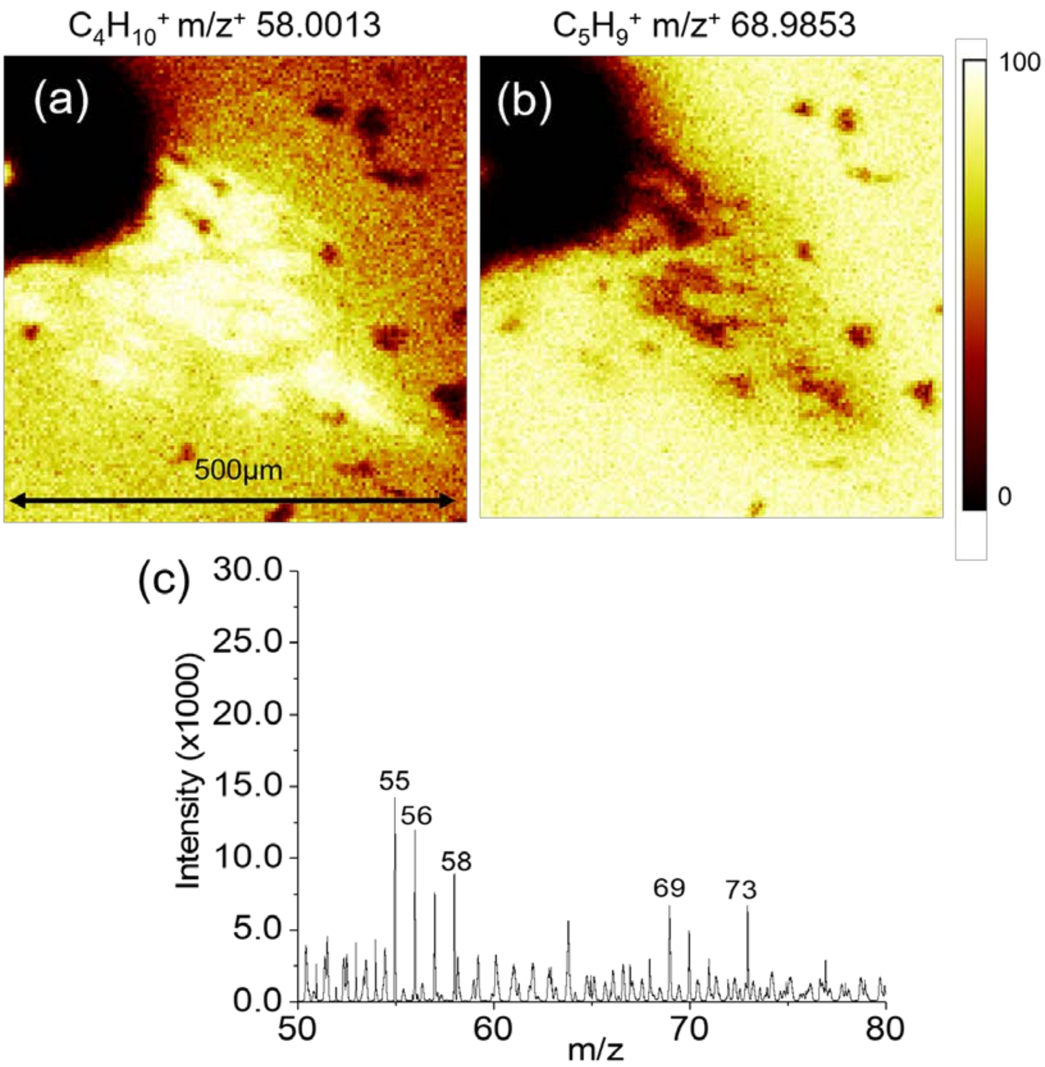

Figure. S5. ToF-SIMS 2D images and a spectrum plot of the graphite droplet stamped on SiN membrane, (**a**) SIMS 2D image of the $C_4H_{10}^+$ and (**b**) $C_5H_9^+$, and (**c**) spectral plot showing graphite fragment ion related peaks.

Fewer significant numbers were used in the spectra due to space constraints.

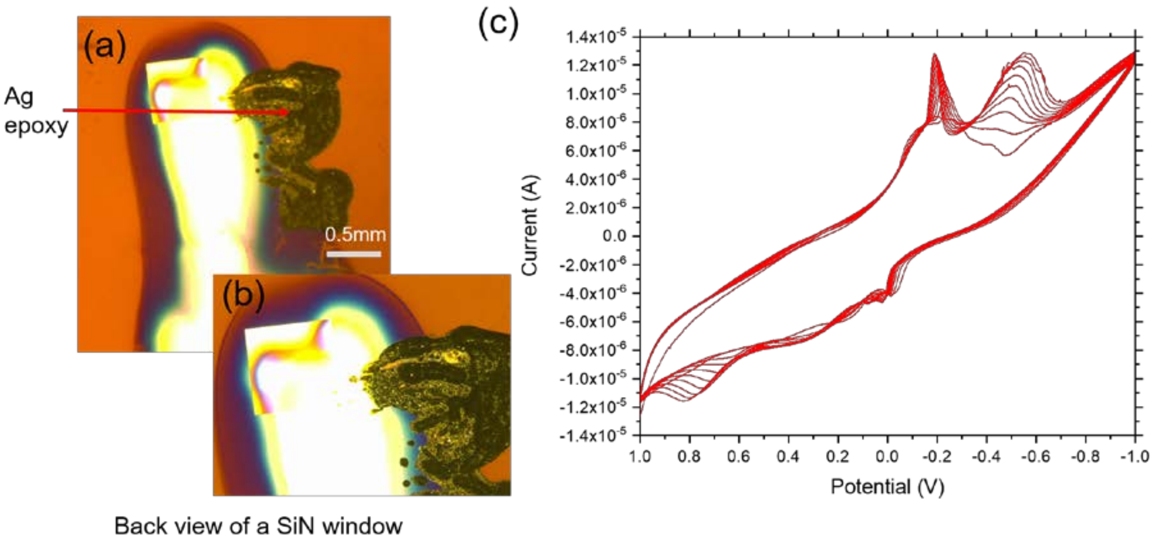

Figure. S6. Optical image of silver epoxy stamped SiN membrane and Cyclic Voltammetry (CV) scan of the silver epoxy control device of 50 x (**a**) and 200 x (**b**) magnification, and (**c**) CV scan plot.

Figures S6a-b show optical microscope images of Ag epoxy stamped SiN and gold electrode boundary of 50x and 200 x magnifications, respectively; and the square structure is a suspended SiN membrane window (0.5 mm x 0.5 mm). Figure S6-c shows the CV scan profile of Ag epoxy applied control device. It has two major peaks at -0.2 V, and -0.5 V during the 1 V to -1V sweep. Then in the -1 V to 1V) sweep, there are peaks at -0.1 and 0.8V. This is a different CV profile compared to the blank control device (Figure S7-c). Figure S6 shows similarities between -0.1 V and 0.8 V during the -1 V to 1 V sweep. In Figure S7c there is a major peak at 0.8 V in the 1 V to -1 V sweep; and there are two peaks at -0.1 V and 0.8V during -1 V to 1 V sweep. This represents a CV profile of the Ag epoxy control device inherent of the blank control device. The new peaks are due to electrochemical reaction products characteristic of Ag epoxy.

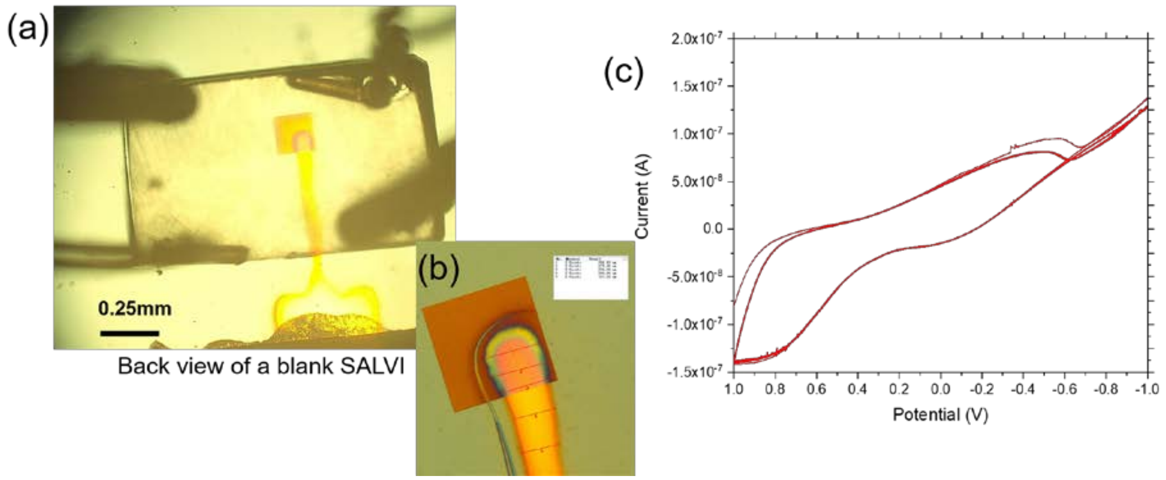

Figure. S7. Optical image of blank SiN membrane with Au electrode and CV scan of the blank control device of 50 x (**a**) and 200 x (**b**) magnification, and (**c**) CV scan plot.

Figure S7c shows CV scan profile of the blank System for Analysis at the Liquid-Vacuum Interface (SALVI) device. Peaks from this plot represent the background characteristic of the device which has similarities to Figures 4-5 of the main text.

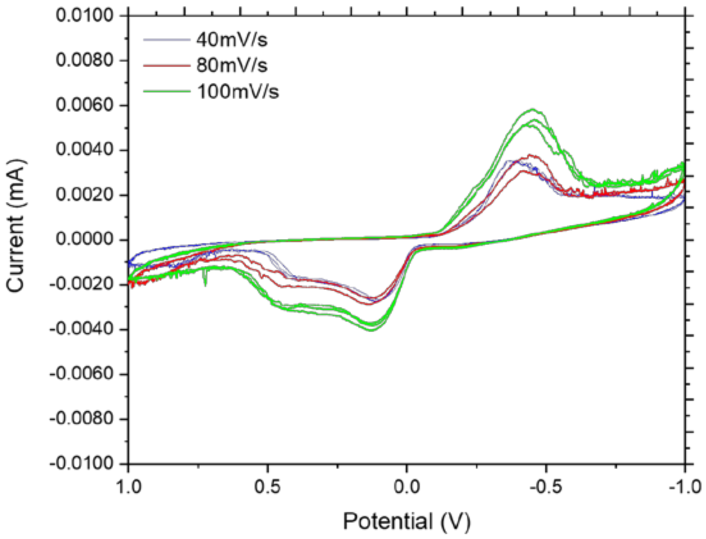

Figure. S8. CV scans of a sequence stamp device with different scan rates, i.e., 40 mV/s, 80 mV/s, and 100 mV/s.

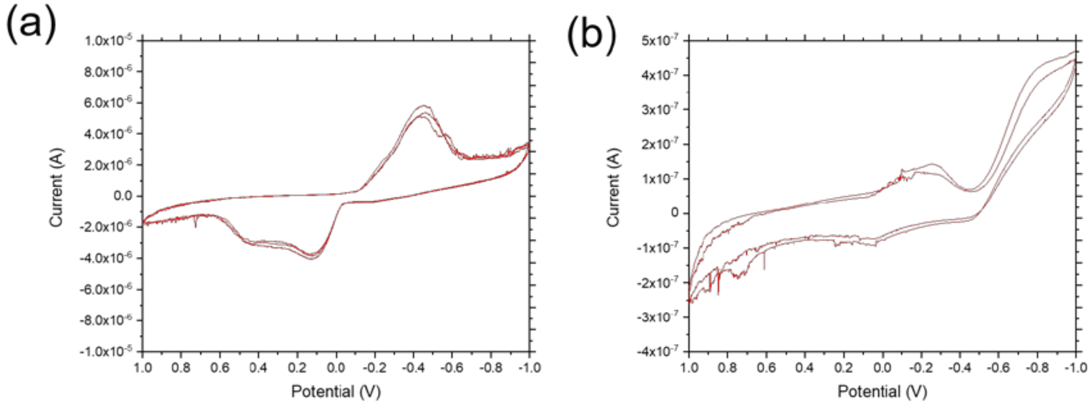

Figure. S9. Comparison of CV scan of the two sequence stamp devices using the scan rate of 100 mV/s: (**a**) the same device as shown in the main test and (**b**) a different device.

Figure S8 shows CV plots of the CeO_2 sequence stamped device using different scan rates (i.e., 40, 80, 100 mV/s) from the same device shown in Figure 4-a. Figure S9 gives a CV comparison between two CeO_2 sequence stamped devices with a scan rate of 100 mV/s, namely, a different device and the device from Figure 4-a. Figure S8 shows the same CV characteristics as in Figure 4-a CeO_2 using the sequence stamped device. Results in Figure S9-b also share similar peak characteristics as those in Figure S9-a, which is same plot as Figure 4-a. Similarity peaks are -0.4 V (sweeping from 1 V to -1 V) and 0.1V (sweeping from -1 V to 1 V). Slight noise is seen in Figure S9-b due to some impurity on the electrode surface which can be improved in future work.

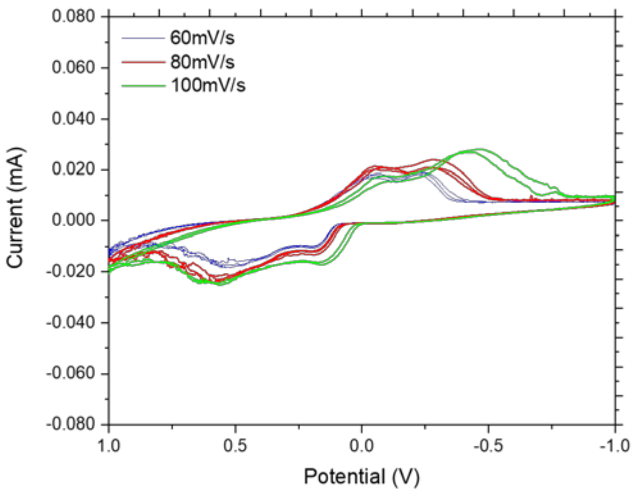

Figure. S10. CV scans of a mix stamp device with different scan rates, i.e., 60 mV/s, 80 mV/s, and 100 mV/s.

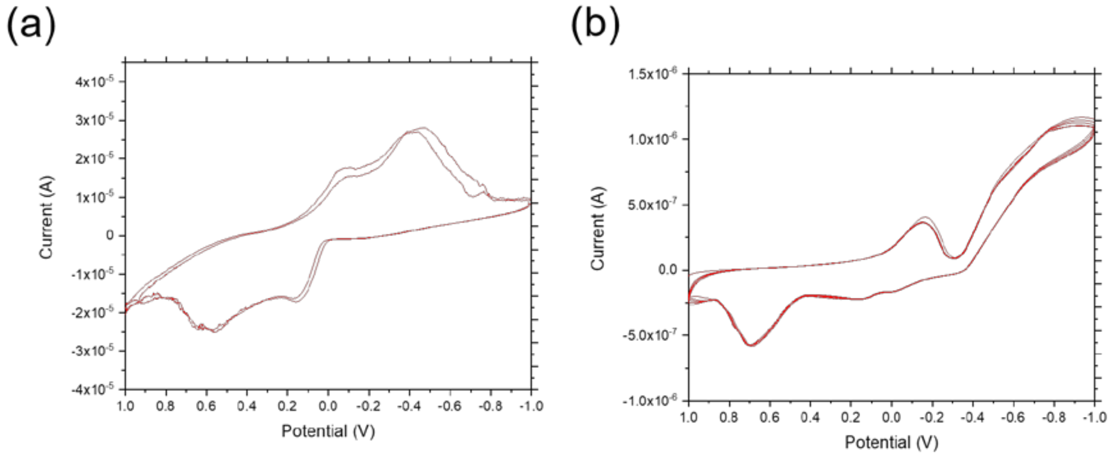

Figure. S11. Comparison of CV scan of the two mix stamp devices using the scan rate of 100 mV/s: (**a**) the same device as shown in the main test and (**b**) a different device.

Figure S10 shows CV plots of the CeO_2 mix stamped device using different scan rates (i.e., 60, 80, 100 mV/s) from the same device shown in Figure 4-a. Figure S11-b gives a CV comparison between two CeO_2 mix stamped devices with the scan rate of 100 mV/s, namely, a different device and the device from Figure 4-a. Figure S10 shows the same CV characteristics as in Figure 4-a CeO_2 using the mix stamped device. Results in Figure S11-b also share similar peak characteristics as those in Figure S11-b, which is same plot as Figure 4-a. Similarity peaks are -0.1 V (sweeping from 1 V to -1 V) and 0.2 V (sweeping from -1 V to 1 V). Slight noise is seen in Figure S11-b due to some impurity on the electrode surface which can be improved in future work. Figure S11-b also shows a slight left shift of 0.55V (sweeping from -1 V to 1 V) to 0.6 V (sweeping from -1 V to 1 V), which can be explained as potential shift due to slight physical difference of nanoparticle applied electrode.

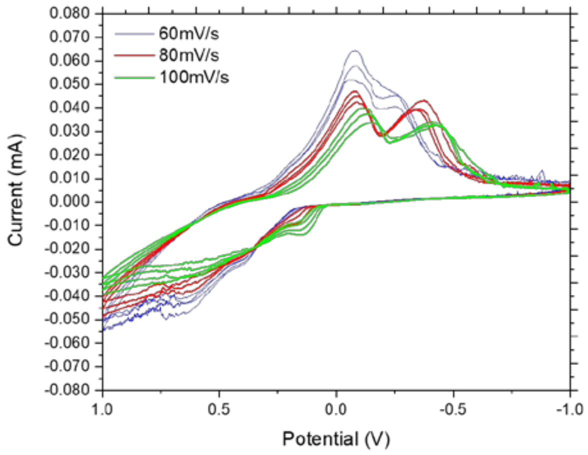

Figure. S12. CV scans of a droplet stamp device with different scan rates, i.e., 60 mV/s, 80 mV/s, and 100 mV/s.

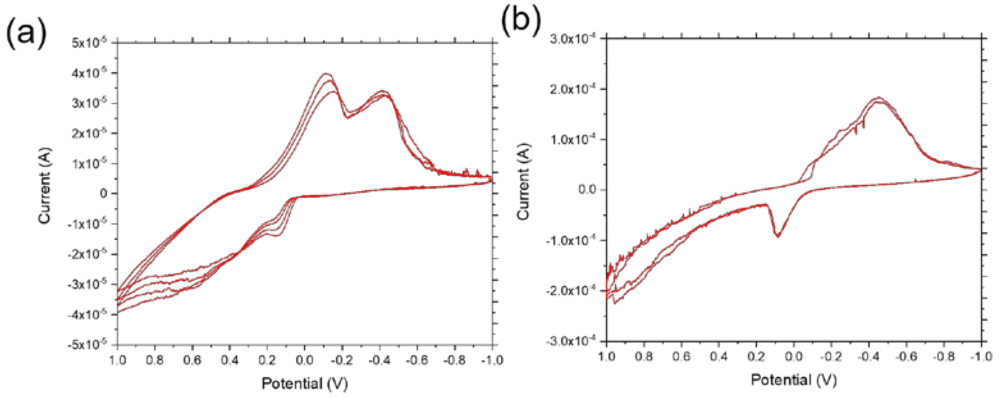

Figure. S13. Comparison of CV scan of the two droplet stamp devices using the scan rate of 100 mV/s: (**a**) the same device as shown in the main test and (**b**) a different device.

Figure S12 shows CV plots of the CeO_2 droplet stamped device using different scan rates (i.e., 60, 80, 100 mV/s) from the same device shown in Figure 4-a. Figure S13-b gives a CV comparison between two CeO_2 droplet stamped devices with the scan rate of 100 mV/s, namely, a different device and the device from Figure 4-a. Figure S12 shows the same CV characteristics as in Figure 4-a CeO_2 using the droplet stamp device. Results in Figure S13-b also share similar peak characteristics as those in Figure S4-a. Figure S13-b also shares similar characteristics with Figure S13-a. The latter is same plot from Figure 4-a. Similarity peaks are -0.12 V, -0.42 V (sweeping from 1 V to -1 V), and 0.1V (-1 V to 1 V).

Reference

1. Smith, T.E., S. McCrory, and M.L. Dunzik-Gougar, *Limited Oxidation of Irradiated Graphite Waste to Remove Surface Carbon-14.* Nuclear Engineering and Technology, 2013. **45**(2): p. 211-218.
2. Deslandes, A., M. Jasieniak, M. Ionescu, J.G. Shapter, C. Fairman, J.J. Gooding, D.B. Hibbert, and J.S. Quinton, *ToF-SIMS characterisation of methane- and hydrogen-plasma-modified graphite using principal component analysis.* Surface and Interface Analysis, 2009. **41**(3): p. 216-224.
